# Natural autoantibodies reactive with glycosaminoglycans in rheumatoid arthritis

**DOI:** 10.1186/ar2507

**Published:** 2008-09-12

**Authors:** Bence György, László Tóthfalusi, György Nagy, Mária Pásztói, Pál Géher, Zsolt Lörinc, Anna Polgár, Bernadett Rojkovich, Ilona Ujfalussy, Gyula Poór, Péter Pócza, Zoltán Wiener, Petra Misják, Agnes Koncz, András Falus, Edit I Buzás

**Affiliations:** 1 Department of Genetics, Cell- and Immunobiology, Semmelweis University, Nagyvarad ter 4, H-1089, Budapest, Hungary; 2 Department of Pharmacodynamics, Semmelweis University, Nagyvarad ter 4, H-1089, Budapest, Hungary; 3 Department of Rheumatology, Semmelweis University, Frankel Leó utca 54, H-1027, Budapest, Hungary; 4 Institute of Enzymology, Biological Research Center, Hungarian Academy of Sciences, Karolina út 29, H-1518, Budapest, Hungary; 5 National Institute of Rheumatology and Physiotherapy, Frankel Leó utca 25-29, H-1023, Budapest, Hungary; 6 Heim Pal Hospital, Ülloi út 86, H-1089, Budapest, Hungary; 7 Research Group for Inflammation Biology and Immunogenomics, Hungarian Academy of Sciences, Nagyvarad ter 4, H-1089, Budapest, Hungary

## Abstract

**Introduction:**

Although natural autoantibodies make up the majority of circulating immunoglobulins and are also present in high numbers in therapeutically used intravenous immunoglobulin preparations, they have received little attention and their precise role remains largely unknown. An increasing awareness of the importance of posttranslational autoantigen modifications and glycobiology led us to explore carbohydrate-reactive natural autoantibodies in patients with rheumatoid arthritis. This study examined systematic antibodies reactive to glycosaminoglycans (GAGs), the carbohydrate components of proteoglycans that are released in large amounts from degrading cartilage.

**Methods:**

To measure antibodies reactive to six different types of GAGs, a specialised ELISA was used in which the carbohydrates were covalently linked to the plastic surface through a 2 nm spacer. Sera from rheumatoid arthritis patients (n = 66), umbilical cord serum samples (n = 11) and adult controls (n = 54) were studied. In order to explore cross-reactivity with microbial antigens, bacterial peptidoglycans and fungal polysaccharides were used. Sera and synovial fluid samples were also tested using a GlycoChip carbohydrate array to characterise individual carbohydrate recognition patterns. We followed a multistep statistical screening strategy for screening GAG-reactive antibodies as predictive disease markers.

**Results:**

While anti-GAG antibodies were absent in the umbilical cord sera, they were readily detectable in adult controls and were significantly elevated in patients with rheumatoid arthritis (p < 0.001). Anti-GAG antibodies showed significant cross-reactivity among different types of GAGs. They also reacted with bacterial peptidoglycans and fungal polysaccharides. Interestingly, anti-chondroitin sulphate C IgM antibody levels showed inverse correlation both with the Disease Activity Score (DAS) 28 scores and C-reactive protein (CRP) levels in rheumatoid arthritis.

**Conclusion:**

The highly abundant and cross-reactive, GAG-specific natural autoantibodies in serum may serve as novel disease-state markers in patients with rheumatoid arthritis.

## Introduction

Rheumatoid arthritis (RA) is a chronic, destructive autoimmune disease of the joints, which affects about 0.5 to 1% of the population [1]. It is characterised by the presence of autoantibodies that are reactive to various target molecules [2,3]. The best known autoantibodies include rheumatoid factor (RF), anti-citrullinated protein antibodies (ACPA) [4-6] and anti-collagen antibodies. Autoantibodies have attracted increasing attention recently and it is estimated that at least 50% of patients with RA have a preclinical phase associated with elevated levels of certain autoantibodies [4-6]. RF, an antibody reactive to the Fc portion of IgG, has been long implicated in the pathogenesis of RA. RF is also produced during the course of the physiological response to various viral and bacterial infections and during certain inflammatory conditions, in order to help eliminate the immune complexes formed [7]. Highly specific RFs are present in RA and may contribute to the joint inflammation, and may help B cells to take up and present various autoantigens [7]. Both RF and ACPA are important prognostic factors in RA.

Serum IgMs are predominantly B1 B-cell-derived natural autoantibodies (NAbs). These polyreactive, low-affinity immunoglobulins are known to represent a first-line defence against infectious agents. They are also known as components of the immunological homunculus, the immune system's built-in self-representation of the body [8]. Some NAbs recognise carbohydrates, but the role of carbohydrate-specific NAbs in RA has not been fully investigated yet.

The present study focuses on NAbs that are reactive to glycosaminoglycans (GAGs), important molecular constituents of both cell surface proteoglycans and large and small proteoglycans of the extracellular matrix of hyaline cartilage. GAGs are released from the degrading cartilage matrix in large amounts during inflammation of the joints. They are composed of repetitive disaccharide units of a hexosamine and hexuronic acid attached through a linker oligosaccharide region to the core protein of proteoglycans. A high number of GAGs are linked to the core protein of cartilage aggrecan. These negatively charged carbohydrates are responsible for the high swelling capacity of cartilage.

Our previous studies demonstrated that in Bagg Albino (BALB/c) mice, human aggrecan (partially depleted in its GAG chains) can provoke a chronic, progressive autoimmune polyarthritis (proteoglycan aggrecan-induced arthritis [PGIA]) that is similar to human RA, and the disease can be transferred to naïve syngeneic mice [9]. We have previously shown that GAG side chains play an important role in the pathogenesis of aggrecan-induced arthritis; although keratan sulphate can mask certain T-cell epitopes, chondroitin-sulphate stubs provoke a strong B-cell response and GAG-specific B cells are important antigen-presenting cells during the development of aggrecan-induced murine arthritis [10]. A high correlation between levels of serum and synovial fluid antibodies reactive to aggrecan and biglycan has been described [11] and may have been due to the presence of shared GAG chains of the two different proteoglycans.

To the authors' knowledge, this is the first study to describe significantly elevated anti-GAG antibody levels in sera of patients with RA and to show cross-reactivity with bacterial and fungal peptidoglycans. Our data suggest that anti-chondroitin sulphate C IgM NAbs may serve as disease-state markers of RA.

## Materials and methods

### Sample selection

Sera from 66 patients with RA (mean age +/- SD 62.5 +/- 9.13 years; range 42 to 88 years; 52 females; 14 males) was examined in this study. Serum and synovial fluid samples from five patients with RA were also tested in the study (three males, two females). All RA patients fulfilled the diagnostic criteria of the American College of Rheumatology (ACR) [12]. Patients were treated in the Department of Rheumatology, Semmelweis University, and the National Institute of Rheumatology and Physiotherapy, both in Budapest, Hungary. Control serum samples (n = 55) representing the natural Caucasian Hungarian population were obtained from the National Traumatology Hospital, Budapest (mean age +/- SD 59.7 +/- 11.6 years; range 31 to 84 years; 43 females; 11 males), and 11 umbilical cord blood samples were obtained from the first Department of Obstetrics and Gynecology, Semmelweis University, Budapest. The clinical and serological data of the patients were documented at the time of venepuncture.

Patients were divided into three groups based on their disease activity score (DAS) 28: those with DAS 28 scores of 3.2 or less were considered to have low disease activity (DAS1); patients with DAS 28 scores of 3.2 to 5.1 were considered to have medium disease activity (DAS2); and patients with DAS 28 score of 5.1 or more were considered to have high disease activity (DAS3) [13]. Serum and synovial fluid samples were stored at -20°C until use. During the entire investigation period we followed the guidelines and regulations of the Helsinki Declaration in 1975, and the experiments were approved by the Ethical Committee of Semmelweis University; all patients or parents of children signed an informed consent form.

### Determination of anti-CCP antibody levels

Serum antibodies reactive to cyclic citrullinated peptide (CCP) were measured with a commercial ELISA (Immunoscan RA Anti-CCP test kit, Eurodiagnistica AB, Malmö, Sweden) according to the manufacturer's instructions.

### Determination of rheumatoid factors

For the determination of IgM and IgG RFs in the sera of patients with RA and controls, we used AUTOSTATTMII RF IgM and IgG kits (Hycor Biomedical GmbH, Kassel, Germany). RFs can interfere with ELISA results by binding to the antigen and then subsequently to the detection antibody giving false-positive results. To rule out this possibility, we used the CovaLink ELISA system (Nunc, Wiesbaden, Germany) to see if RFs could bind to GAGs. We used IgM and IgG RFs provided by the AUTOSTATTMII RF IgM and IgG kits (Hycor Biomedical GmbH, Kassel, Germany). We tested a concentration range of RF IgM of 0.128 to 80 IU/mL and IgG 0.0512 to 32 IU/mL, but could not find any evidence of RF reactivity to any of the GAGs.

### Determination of serum C-reactive protein levels

C-reactive protein (CRP) levels were determined with a Full Range CRP turbidimetric assay (Randox Laboratories Ltd. Crumlin, County Antrim UK) was performed using Olympus AU600 biochemistry analyser (Olympus Medical Systems Europa GmbH, Hamburg, Germany) according to the manufacturer's instructions.

### Detection of carbohydrate-specific antibodies

The GAGs used in this study included chondroitin sulphate A (CSA), chondroitin sulphate B (CSB), chondroitin sulphate C (CSC), keratan sulphate (KS), low molecular weight heparin sulphate (HS) and hyaluronic acid (HA) (all purchased from Sigma-Aldrich Ltd. St. Louis, MO). The problem of poor carbohydrate binding to polystyrene surfaces was solved by the use of a CovaLink ELISA, a system that uses a 2 nm spacer arm rendering surface-bound carbohydrates accessible by antibodies [14]. Briefly, carbohydrate antigens were covalently bound to the surface of the CovaLink plates (Nunc, Wiesbaden, Germany) using 1% 1-(3-dimethylaminopropyl)-3-ethylcarbodiimid (1% EDC, Merck Whitehouse Station, NJ) at 1 μg/well. Plates were incubated for two hours at 37°C and then overnight at room temperature. Blocking was carried out using 1% PBS-BSA-Na azide for two hours at room temperature. Sera and synovial fluid samples were used at a 1:100 dilution (a concentration selected after preliminary experiments). HRP-conjugated anti-human IgM and anti-human IgG (both from Sigma-Aldrich, St. Louis, MO) were used as secondary antibodies at 1:50000 and 1:30000 dilutions, respectively. Orto-phenylene-diamine (Sigma-Aldrich, St. Louis, MO) and 0.33% hydrogen peroxide were added and the absorbance was detected at 492 nm. For each plate standard curves were drawn using known amounts of nonconjugated human IgG and IgM (both from Sigma-Aldrich, St. Louis, MO).

To test if the presence of RF in human sera interferes with the carbohydrate-specific ELISAs, we collected RF from randomly selected RA serum samples (n = 6). We coupled heat aggregated (63°C, 30 minutes) human IgG (Sigma Aldrich, St. Louis, MO) to cyanogen bromide activated Sepharose 4B (Sigma Aldrich, St. Louis, MO) at 5 mg/ml gel. For two hours 250 μl sera were incubated with continuous stirring in the presence of 500 μl human IgG-coupled resin. The removal of RF was confirmed using the AUTOSTATTMII RF kit, and carbohydrate ELISAs were repeated with RF-containing and RF-free serum pairs. Removal of the RFs did not influence the carbohydrate recognition pattern.

### Determination of total IgM and IgG levels

We used the CovaLink ELISA system to determine the total IgM and IgG levels of the patients and controls. For each plate standard curves were drawn using known amounts of nonconjugated human IgG and IgM.

### Inhibition of binding of anti-GAG antibodies

GAGs, bacterial peptidoglycans, a fungal polysaccharide and a weakly anionic exchanger resin (Duolite C433, Sigma-Aldrich, St. Louis, MO) were used to inhibit the binding of GAG-reactive antibodies. We tested peptidoglycans from *Escherichia coli, Staphylococcus aureus*, *Bacillus subtilis *and the fungal polysaccharide, Zymosan (all purchased from Invitrogen, Carlsbad, CA). Serum samples, diluted to 1:100 with PBS-Tween, were preincubated for two hours at 37°C with 0.5 μg/μl, 5 μg/μl and 50 μg/μl peptidoglycans, Zymosan and GAGs. All three antigens were used in three different concentrations in the same volume. To 1.5 ml serum diluted to 1:100 with PBS-Tween, 20 mg of Doulite C433 (Sigma) was added. After two hours of preincubation at 37°C, the resin was pelleted and the supernatant was used in CovaLink ELISA.

### Digestion of aggrecan with glycosidases

Bovine aggrecan was purchased from Sigma-Aldrich. Human aggrecan monomers were purified from human newborn cartilage samples. The use of human cadaver cartilage was approved by the Institutional Review Board of Semmelweis University. Aggrecan monomers were isolated as previously described [15]. Briefly, cartilage samples were dissected and extracted at 4°C in 4 M guanidine hydrochloride and 50 mM sodium acetate at a pH of 5.8 for 48 hours. High buoyant density aggrecan monomers were prepared by dissociative cesium chloride density gradient ultracentrifugation. Aggrecan was digested with either β-galactosidase from jack bean (Sigma-Aldrich, St. Louis, MO) or hyaluronidase from sheep testis (Sigma-Aldrich, St. Louis, MO). Proteoglycans were digested with 100 U/mg of β-glucuronidase in 0.15 M citrate-phosphate buffer, at pH 4.3, and 240 U/mg of hyaluronidase in 0.2 M NaCl-acetate buffer, at pH 5.0, for 24 hours at 37°C in the presence of protease inhibitors (10 mM EDTA, 2 mM PMSF, 2 mM iodoacetamide and 5 μg/ml pepstatin A) (all purchased from Sigma-Aldrich, St. Louis, MO). Digested proteoglycans were stored at -20°C until used. Both native and digested human and bovine aggrecans were used to coat the conventional ELISA plates (Nunc Maxisorp, Nunc, Wiesbaden, Germany) 0.2 μg protein/well, and were incubated for two hours at 37°C. Blocking and incubation with primary and secondary antibodies were carried out the same way as described earlier for the CovaLink ELISA system.

### Glycochip

IgGs of serum and synovial fluid samples were labelled with Alexa Fluor 350-conjugated antihuman IgG antibody (Fab fragment) (Zenon Human IgG Labeling Kits, Molecular Probes Inc. Invitrogen (Invitrogen Corporation, Carlsbad, CA) according to the manufacturer's instructions. Briefly, 1 μg Fab was used to label 1 μg IgG in TBS. Labelled IgGs 10 μl/well were applied to the Glycochip (Glycominds Ltd., Lod, Israel) at a concentration of 10 μg/ml of IgG. The Glycochip included the following surface-bound carbohydrate structures: Gal (a); Gal (b); Gal (b1-3) [GlcNAc (b1-6)] GalNAc (a); Gal (b1-3) GalNAc (a); Ab3GNb Gal (b1-3) GlcNAc (b); Gal (b1-4) Glc (b); Gal (b1-4) GlcNAc (a); Gal (b1-4) GlcNAc (b); Gal (b1-6) Gal (b); GalNAc (a); GalNAc (b); Fuc (a); Fuc (a1-2) Gal(b); Fuc (a1-2) Gal(a); Fuc (b); Glc (a); Glc (a1-4) Glc (a); Glc (a1-4) Glc (b); Glc (b); Glc (b1-4) Glc (b); Glc (b1-4) Glc (b1-4) Glc (b); GlcNAc (a); GlcNAc (b); GlcNAc (b1-3) GalNAc (a); GlcNAc (b1-4) GalNAc (b); GlcNAc (b1-6) GalNAc (a); L-Rha (a); GalA (b); Man (a); Man (a1-3) Man (a); Man (b); Man (b1-4) Glc (b); Neu5Ac (a); L-Araf (a), GlcA (b); Xyl (a); Xyl (b).

The carbohydrate array was incubated for two hours at 37°C, and then washed three times with a buffer containing 2 M NaCl, 0.04 M MgSO4 and 0.5% Tween 20. In order to reduce the background, plates were soaked for 10 to 15 minutes in the same buffer after the last washing step. After washing, 10 μl of TBS was added to the plate before measuring fluorescence on a Perkin Elmer Victor II spectrofluorimeter. Excitation wavelength was 355 nm; emission was detected at 460 nm.

### Immunohistochemistry

Normal adult human cadaver cartilage was cut by cryostat and mounted onto SuperFrost (Thermo Fisher Scientific, Waltham, MA) slides. Specimens were fixed immediately by alcohol/acetone for five minutes, and the non-specific binding sites were blocked by 5% BSA (Sigma Aldrich, St. Louis, MO) in PBS for 45 minutes at room temperature in a humid chamber. Specimens were washed in PBS, and incubated with RA serum (1:25) or with serum that was preincubated with 2 mg/ml or 4 mg/ml CSC for 60 minutes at room temperature. Specimens were washed three times in PBS, and incubated with the fluorescein isothiocyanate-labelled anti-human immunoglobulin (1:100 dilution, Sigma-Aldrich, St. Louis, MO) for 45 minutes at room temperature, and then washed three times in PBS. Cover slips were mounted on the slides using PBS-glycerin (1:1) as a mounting medium. The slides were analysed in a Bio-Rad MRC 1024 confocal laser scanning microscope (Nikon Instruments Inc. Melville, NY) equipped with a krypton/argon mixed gas laser. Excitation was carried out at 480 nm and 540 nm. The fluorescence intensities of each experiment were normalised to the negative control (from which the primary antibody was omitted). All negative controls demonstrated negligible background fluorescence.

### Statistical analysis

#### ELISA assays

We fitted a three-parameter logistic curve to calibrate data and the unknown anti-GAG sample concentrations were determined from the fitted calibration curve. In the case of CCP assays, we subtracted the blank (zero concentration) values from the standard dilution series data and serum anti-CCP concentrations were obtained by linearly interpolating this standard curve. We found that the distribution of anti-GAG antibodies and CRP concentrations were highly skewed to the right. Therefore, we logarithmically transformed them, and all statistical computations were carried out using this data.

#### ANOVA

A three-factor one-way analysis of variance (ANOVA) model was used for overall comparisons of sera of patients compared with sera of adult controls and umbilical cord sera. The dependent variable was the logarithmically transformed antibody concentration. The independent factors were: the indicator variable named "Status" showing the group membership (like control or patient), a variable named "anti-GAG" with levels of GAG, and a third variable named "IgX" showing that the antibody was of IgM or IgG type. This is a multiplicative model because anti-GAG concentrations were logarithmically transformed, and the null hypothesis was that the relative increase due to factor "Status" was not significantly different from one.

#### Screening of GAG-reactive antibodies as predictive disease markers

We followed the screening strategy advised by Harrell to find which of the anti-GAG antibodies would be the best disease specific marker(s) [16]. The strategy is based on screening variables for their predictive power and carrying out confirmatory tests only with the promising candidates. The advantage of this strategy is that the problem of multiple testing and the statistical difficulties due to co-linearity of the variables can be avoided or greatly reduced. Principal component analysis is an alternative way of reducing the dimensionality of the data when the variables are highly co-linear, but it has the disadvantage that the resulting components are sometimes difficult to interpret.

As a first step of our screening procedure, stepwise logistic regression was used to select those anti-GAG antibody types that best discriminated the patient group from the controls. In the second step of the screening, stepwise ordinal regression was used to select those anti-GAG antibodies that had the highest predictive power to correctly categorise patients, according to their disease activity. In the present case the dependent variable was the DAS score (1, 2 and 3), while the independent variables were the concentrations of the molecules that were selected in the first step. In both procedures candidate variable selection stopped when no step decreased further than the Akaike's information criterion [17]. As a final step, we used ANOVA followed by Tukey's post-hoc test to statistically confirm the relation between the anti-GAG antibody concentration and disease status. All statistical computations were carried out using Splus (Release 6.1, Professional version, Insightful, WA) with additional procedures StepAIC and polr from the Mass library [17].

## Results

### Anti-GAG antibody concentrations of serum samples

Table 1 lists the concentrations of GAG-reactive IgM and IgG antibodies in neonatal serum samples, in sera of adult controls and in sera of patients with RA. Anti-GAG antibodies were almost undetectable and uniformly extremely low in all umbilical cord samples compared with adult controls. In the case of IgM, this was expected, because the total IgM in the umbilical blood serum is reported to be very low [18]. The difference between serum anti-GAG antibody levels of controls and umbilical cord samples was significant (three-way ANOVA, F = 110.70; DF = 1.77; p < 0.001). F indicates F-statistics, DF indicates degree of freedom. Initial statistical analysis showed that the anti-GAG concentrations were significantly higher in the sera of RA patients as compared with adult controls. The mean antibody concentrations were two to four times higher in patients than in controls (Table 1). The difference was highly significant (three-factor ANOVA, F = 30.17; DF = 1.11; p < 0.001), and independent from the type of antigen and the type of antibody. In terms of ANOVA model, the interaction of "GAG" with "Status" and "IgX" with "Status" were not significant (F = 0.59, N.S. and F = 0.72, N.S. respectively).

**Table 1:** Anti-glycosaminoglycan antibody concentrations

**Antibody (μg/mL)**	**Umbilical cord serum**	**Control serum**	**RA patients serum**
	**Mean ± SEM**	**Mean ± SEM**	**Mean ± SEM**
**Anti-chondroitin sulphate A IgM**	1.63 ± 0.3	750.95 ± 343.1	1225.27 ± 354.2
**Anti-chondroitin sulphate B IgM**	1.36 ± 0.2	608.00 ± 302.3	1300.61 ± 389.2
**Anti-chondroitin sulphate C IgM**	1.73 ± 0.4	673.70 ± 305.7	1964.34 ± 461.7
**Anti-keratan sulphate IgM**	1.63 ± 0.2	275.11 ± 184.5	836.87 ± 321.3
**Anti-heparan sulphate IgM**	2.27 ± 0.4	1115.91 ± 383.0	2605.36 ± 516.8
**Anti-hyaluronic acid IgM**	1.27 ± 0.2	4.32 ± 0.9	14.18 ± 4.5
			
**Anti-chondroitin sulphate A IgG**	1.54 ± 0.4	203.09 ± 89.1	872.79 ± 310.7
**Anti-chondroitin sulphate B IgG**	1.77 ± 0.8	508.07 ± 215.9	1551.62 ± 417.2
**Anti-chondroitin sulphate C IgG**	1.27 ± 0.1	241.53 ± 176.3	717.86 ± 273.3
**Anti-keratan sulphate IgG**	4.91 ± 4.3	1871.63 ± 481.1	2704.11 ± 525.2
**Anti-heparan sulphate IgG**	4.05 ± 3.8	1066.59 ± 326.7	2689.71 ± 515.8
**Anti-hyaluronic acid IgG**	1.09 ± 0.2	960.93 ± 365.3	1227.82 ± 379.3

Mean antibody concentrations are expressed in μg/mL. The difference between serum anti-glycosaminoglycan (GAG) antibody levels of controls and umbilical cord samples was significant (three-way analysis of variance [ANOVA], F = 110.70; DF = 1.77; p < 0.001). The difference between RA patients and adult controls was also highly significant (three-factor ANOVA, F = 30.17 DF = 1.11 p < 0.001), and independent from type of the antigen and from the type of the antibody as well.

The ratio of anti-CSC and total IgM level was mean 4.68 ± 3.15%, range 0.17 to 21.33% in the patient group, whereas the ratio of anti-CSC and total IgG level was mean 0.83 ± 0.73%, range 0.1 to 14.29%. In the control group the ratio of anti-CSC and total IgM was mean 6.59 ± 3.04%, range 1.77 to 22.97%, while the ratio of anti-CSC and total IgG was mean 1.57 ± 1.36%, range 0.13 to 20.58% (data not shown).

### Synovial fluid anti-GAG antibody concentrations

**Figure 1:**
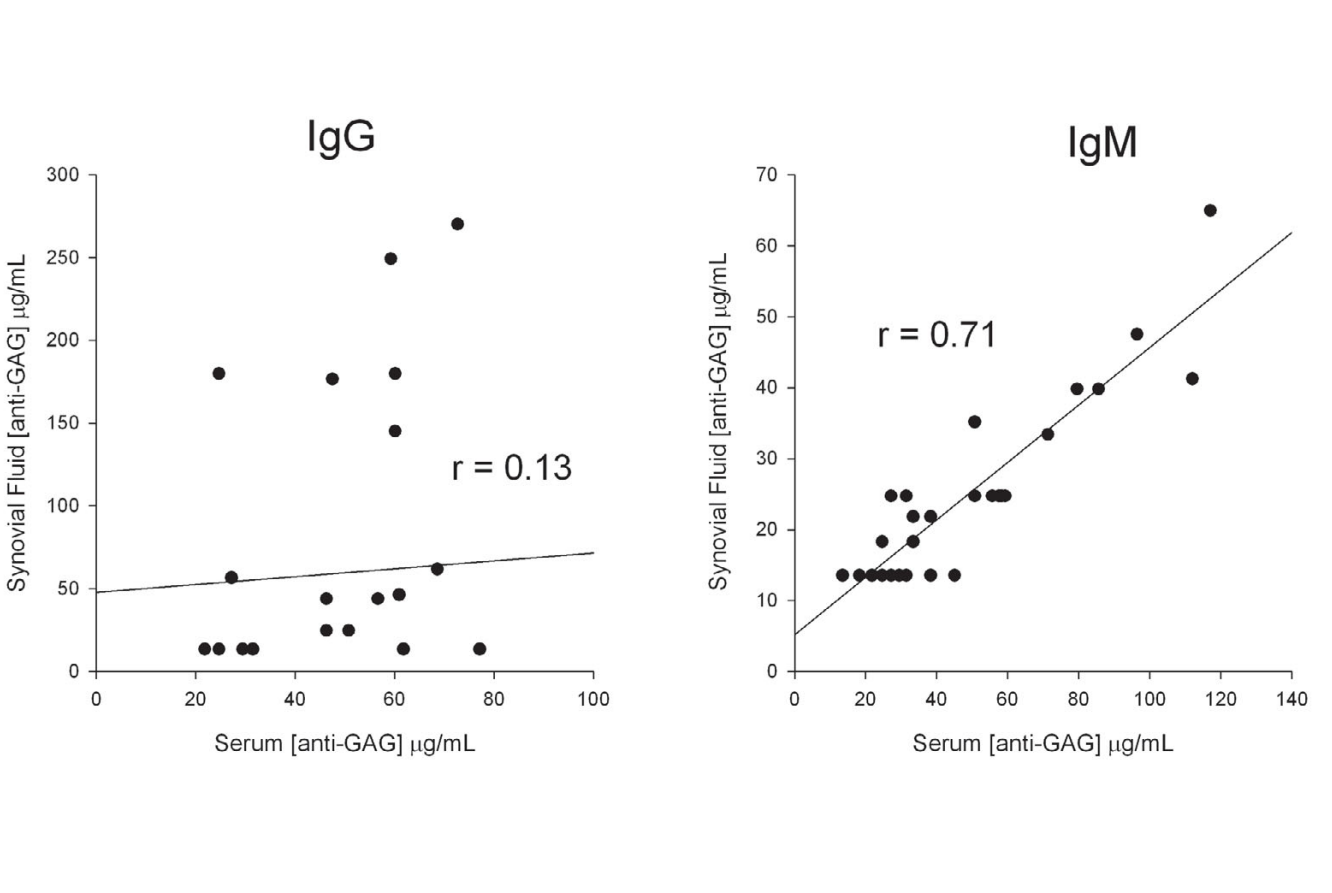
Correlation between serum and synovial fluid anti-glycosaminoglycan (GAG) IgM and IgG antibodies in rheumatoid arthritis (RA). Note the strong and statistically significant correlation (p < 0.001) between the corresponding IgM anti-GAG concentrations in serum and synovial fluid samples. Such a relation could not be demonstrated for IgG antibodies (r = 0.13, N.S).

Anti-GAG concentrations were higher in the serum than in synovial fluid (data not shown). This difference was highly significant (three-factor ANOVA, p < 0.001). There was also significant correlation between serum and synovial fluid IgM antibody concentrations (r = 0.71, p < 0.001) but such a relation could not be demonstrated for IgG antibodies (r = 0.13, N.S. Figure 1).

The ratios of synovial fluid/serum antibodies for IgM antibodies were as follows: CSA 0.15 ± 0.04; CSB 0.23 ± 0.06; CSC 0.37 ± 0.19; KS 0.25 ± 0.10; HS 0.26 ± 0.13; and HA 0.75 ± 0.3. The ratios of synovial fluid/serum antibodies for IgG antibodies were as follows: CSA 4.22 ± 6.53; CSB 1.75 ± 2.34; CSC 19.4 ± 30.84; KS 4.34 ± 6.46; HS 8.10 ± 9.40; and HA 1.32 ± 1.63 (data not shown).

### Anti-GAG antibodies as disease markers

**Figure 2:**
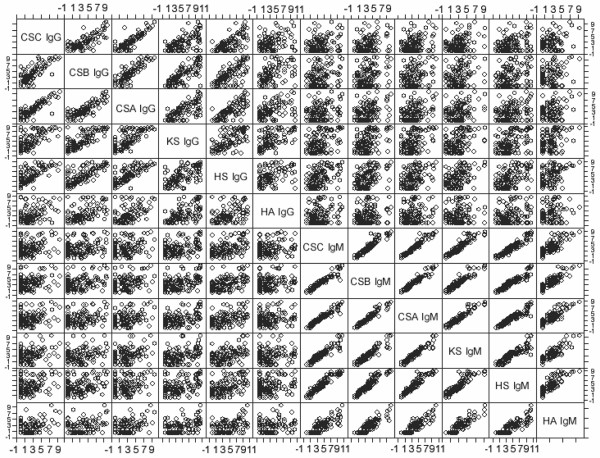
Scatter plot of anti-glycosaminoglycan (GAG) antibody concentrations. Log-transformed antibody concentrations were plotted against each other. Figure 2 suggests that there is a strong relation between anti-GAG antibody concentrations, particularly among IgM molecules. This observation has been confirmed by detailed statistical analysis, and the lowest value of the correlation coefficients between anti-GAG IgM antibodies was 0.86. The concentrations are expressed in μg/mL.

In the initial analysis discussed above, patients were treated as a homogenous group regardless of disease activity at the time of serum sampling. However, RA is a fluctuating disorder and biological markers show that a strong dependence from the functional status of the patient [19]. The initial statistical analysis indicated that anti-GAG antibodies can be useful biomarkers, but it was unknown if they were disease-specific or disease-state-specific biomarkers. A disease-specific biomarker correctly differentiates patients from controls, whereas a disease-state-specific marker separates patients according to their status. To find out if they were they were disease-specific or disease-state-specific biomarkers, we categorised the patients by their DAS 28 scores [13,20]. The relation between anti-GAG concentrations and DAS 28 scores was investigated by the two-step screening procedure described above. In brief, the goal of our screening procedure was to find one or more anti-GAG antibody species from the measured 12 that carry the same biological information as the whole data set. To find such representing variables is only a reasonable strategy in a case when the variables strongly correlate with each other. As Figure 2 shows, anti-GAG antibody concentrations positively correlate with each other. This correlation was particularly strong among IgM molecules, with the correlation coefficient in all cases being higher then 0.8. Principal component analysis (results not shown) also indicated that two linear combinations of the IgM and IgG concentrations could explain 84.5% of the total variance. Therefore, selection of a representative anti-GAG antibody molecule was a statistically reasonable approach.

**Figure 3:**
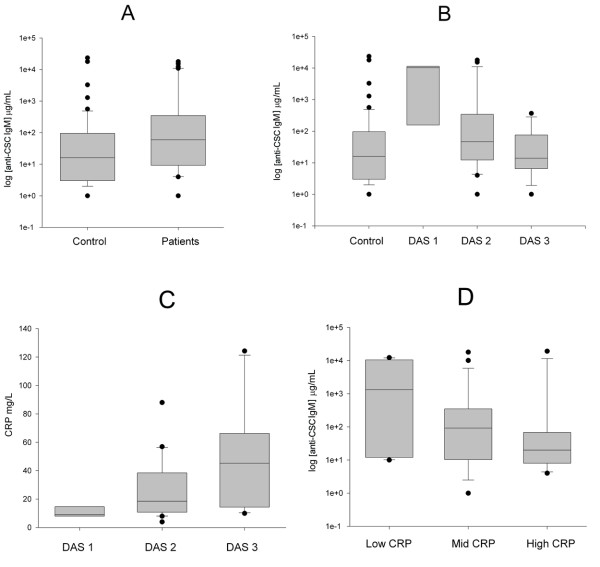
Correlation of anti-chondroitin sulphate C IgM levels with the disease and activity markers in rheumatoid arthritis. **(a) **Box plots of anti-chondroitin sulphate (CS) C IgM concentrations groups of controls and patients with rheumatoid arthritis (RA). The logarithm of the anti-CSC IgM concentration is plotted. The lines inside the boxes denote the medians. The boxes mark the interval between the 25th and 75th percentiles. The ends of the vertical lines or whiskers indicate the minimum and maximum data values, unless outliers are present in which case the whiskers extend to a maximum of 1.5 times the interquartile range. The difference between the controls (n = 55) and RA patients (n = 66) is statistically significant (p < 0.02, F-test). **(b) **Box plots of anti-CSC IgM concentrations in groups of controls and the patients with RA. Patients are stratified according to disease activitity scores (DAS) 28. Patients classified to DAS 1 group (n = 6) have significantly higher anti-CSC IgM concentrations compared with controls and to patients in the DAS 2 (n = 22) and 3 (n = 18) categories. No other significant differences have been found (p < 0.05, post-hoc Tukey's test). Result suggests that anti-CSC IgM is not a disease, but a state dependent marker. **(c) **Comparison of C-reactive protein (CRP) levels in patients according to their DAS scores. On the vertical axis the logarithm of CRP concentration is plotted. The only significant difference was found between groups DAS 1 and 3 (p < 0.05, post-hoc Tukey's test). **(d) **Box plots of anti-CSC IgM concentrations in groups of controls and patients with RA. Patients were stratified according to their CRP values into three subgroups: those having low, moderate or high CRP values (n = 17, n = 16 and n = 16, respectively). The anti-CSC IgM titre decreases when CRP concentration increases; the difference between the low and high CRP group is statistically significant (p < 0.05, post-hoc Tukey's test).

In the first step of the screening, stepwise logistic regression selected five anti-GAG antibody types as being potentially good RA disease markers. The selected five anti-GAG antibodies were: anti-KS IgG, anti-HS IgG, anti-CSC IgM, anti-CSB IgM and anti-HA IgM. In the second step, we used stepwise ordinal regression to find those anti-GAG antibodies that could distinguish patients with different DAS scores. From the five possible candidates selected in the first step, four were eliminated in the second step, and only anti-CSC IgM remained as a possible candidate. We confirmed this hypothesis with ANOVA followed by a Tukey's post-hoc test (Figure 3a). The anti-CSC IgM concentration was significantly higher in RA patients compared with the controls (F = 6.17, DF = 1,93, p < 0.02), but anti-CSC IgM is not a disease-specific maker, but a disease-state marker (Figure 3b). Its value is significantly higher in patients with RA when the disease is inactive (DAS 28 <3.2) compared with controls and patients in the active disease state as well (p < 0.05, ANOVA followed by Tukey's post-hoc test). Conversely, there was no significant difference between controls and patient who were in the active disease period.

A similar relation was found when we analysed the connection between disease activity, CRP and anti-CSC IgM concentrations. CRP significantly increases with increasing DAS scores (F = 4.64, DF = 2.34, p < 0.02) (Figure 3c). Therefore, we expected an inverse relation between disease activity and anti-CSC IgM. To demonstrate this relation, we divided patients having low, moderate and high-CRP concentrations into three categories that contained almost equal number of patients. An inverse relation does exist, although anti-CSC IgM is only significantly different between the low and moderate-CRP groups (F = 3.65, DF = 2.45, p < 0.05) (Figure 3d). Based on these results, we again compared RA patients with controls using all measured anti-GAG concentrations, but now patients were stratified by their DAS scores. The results confirmed our results. The three-way ANOVA model, applied previously, indicated a highly significant difference between patients with low (F = 0.3 DF = 1.71, p < 0.001) and moderate DAS scores (F = 33.35, DF = 1.91). But there was no difference between controls and patients with DAS scores of 5.1 or more (F = 0.22, DF = 1.79).

### The relation between RFs and anti-GAG antibodies

We measured IgG and IgM RF levels of serum samples and found elevated RF levels in patients with RA compared with controls. As expected, the differences were highly significant for both IgG and IgM RFs (unpaired t-test, p < 0.001). We found low-correlation coefficients for erythrocyte sedimentation rate and CRP values of patients with RA. The correlation coefficients between anti-CCP and RF were 0.211 and 0.214 IgG and IgM, respectively. Although we found very low correlation coefficients in the case of certain antibody levels (such as CSC-IgG, CSB-IgG or CSA IgG), others (such as CSC IgM, CSA IgM or HS-IgM) showed relatively high positive correlation. The highest correlation coefficients were found between CSC-IgM and RF concentrations in the case of both IgG and IgM RFs (in RA patients r = 0.384 and r = 0.388 for IgG and IgM RFs, respectively). This might be considered as a further indication of the significance of IgM anti-CSC levels in RA. As expected, the dependence of RF concentrations from DAS scores showed the same pattern as CSC IgM (data not shown).

### The relation between anti-CCP and CSC-IgM antibody levels

Anti-CCP antibodies are well-established prognostic and diagnostic biomarkers in RA. Similar to the findings of others [21], in our study the anti-CCP concentrations did not correlate with the DAS 28 scores (one-way ANOVA, F = 1.24, DF = 2.34, N.S.). Furthermore, there was no correlation between the anti-CCP and CSC-IgM concentrations (r = 0.08, N.S.).

### Anti-GAG antibodies cross-react with other types of GAGs as well as with peptidoglycans and a fungal polysaccharide

To confirm cross-reactivity of anti-GAG antibodies, we analyzed *in vitro *cross inhibition of antibody binding among the various GAG types as well as between GAGs and peptidoglycans or fungal polysaccharides. In line with the very high correlation of antibody levels reactive to different types of GAGs that we found in this study, in systemic inhibition assays we detected broad cross-reactivity among different types of GAGs (data not shown). Surprisingly, we also detected inhibition of binding of antibodies to GAGs by *B. subtilis *peptidoglycan. RA serum IgM was 3.1%, 23.0% and 32.3%; RA serum IgG was 9.3%, 20.6% and 39.4%; control serum IgM was 1.4%, 9.7% and 12.5%; control serum IgG was 22.1%, 22.1% and 33.8%, when using 0.5 μg/μl, 5 μg/μl and 50 μg/μl inhibitor molecules, respectively. We could also identify similar IgM cross-reactions between GAGs and Zymosan in the case of RA serum antibodies (CSA was 2.9%, 10.7% and36.4%; CSB was 11.8%, 19.7% and 40.5%; CSC 12.6%, 17.5% was 48.3%; KS was 11.8%, 23.5% and 50.6%; HS was 4.5%, 11.7% and 22.1%; HA was 15.0%, 207% and 33.0% inhibitions were detected using 0.5 μg/μl, 5 μg/μl and 50 μg/μl inhibitor molecules, respectively).

### Inhibition of binding of anti-GAG antibodies by anionic resin

There was a dramatic decrease in binding of antibodies to GAGs when the serum samples were pre-incubated with the Duolite weakly acidic exchanger resins. We could inhibit RA serum IgM antibodies to bind to CSA, CSB, CSC, KS, HS and HA by Duolite (58.98% ± 0.88%, 59.56% ± 1.53%, 60.77% ± 2.17%, 59.93% ± 0.14%, 64.80% ± 1.76%, 45.95% ± 181.42%, respectively). It is important to note that the weakest inhibition was detected in the case of HA, while the strongest inhibition was seen in the case of HS, GAGs that have the lowest and highest number of negative charged groups, respectively. Doulite could also inhibit binding of IgG type anti-GAG antibodies to CSA, CSB, CSC, KS, HS and HA (70.85% ± 0.05%, 58.57% ± 1.57%, 61.84% ± 0.60%, 64.49% ± 1.19%, 63.25% ± 2.1%, 62.60% ± 0.66%, respectively).

### Antibody recognition of glycosidase-digested human cartilage aggrecan

Glycosidase activity may be elevated within the joints during inflammation and microbial infections [22]. Hyaluronidase digestion of aggrecan increased RA serum and synovial fluid IgM reactivity by 28% (p < 0.05) and 42% (p < 0.001), respectively. Similarly, digestion with β-galactosidase increased RA serum and synovial fluid IgM reactivity to aggrecan by 21% (p < 0.05) and by 22% (p < 0.01), respectively. On the contrary, neither of the glycosidase cleavages affected reactivity of IgM-type control serum antibodies (data not shown). RA synovial fluid, but not serum-derived IgGs showed significantly enhanced reactivity after hyaluronidase-cleavage (31%, p < 0.05). Even IgGs from controls reacted 28% stronger with hyaluronidase-digested aggrecan than with the undigested one (p < 0.05), but their reactivity to β-galactosidase-digested aggrecan was not enhanced (data not shown).

### Determination of carbohydrate recognition patterns

To determine individual carbohydrate recognition patterns, we used a Glycochip system. IgGs of sera of five patients with RA, synovial fluid samples of five RA patients and of five sera of control adult individuals were tested on Glycochip plates. Although there were some carbohydrates to which we could detect higher reactivity in some of the patients with RA than in the controls (such as Gal(a), Man(a) and GlcA (b), Gal(b1-3) [GlcNAc(b1-6)] GalNAc (a)), we could not identify a clear carbohydrate recognition pattern specific for RA (data not shown).

### Binding of carbohydrate-specific antibodies to cartilage matrix

**Figure 4:**
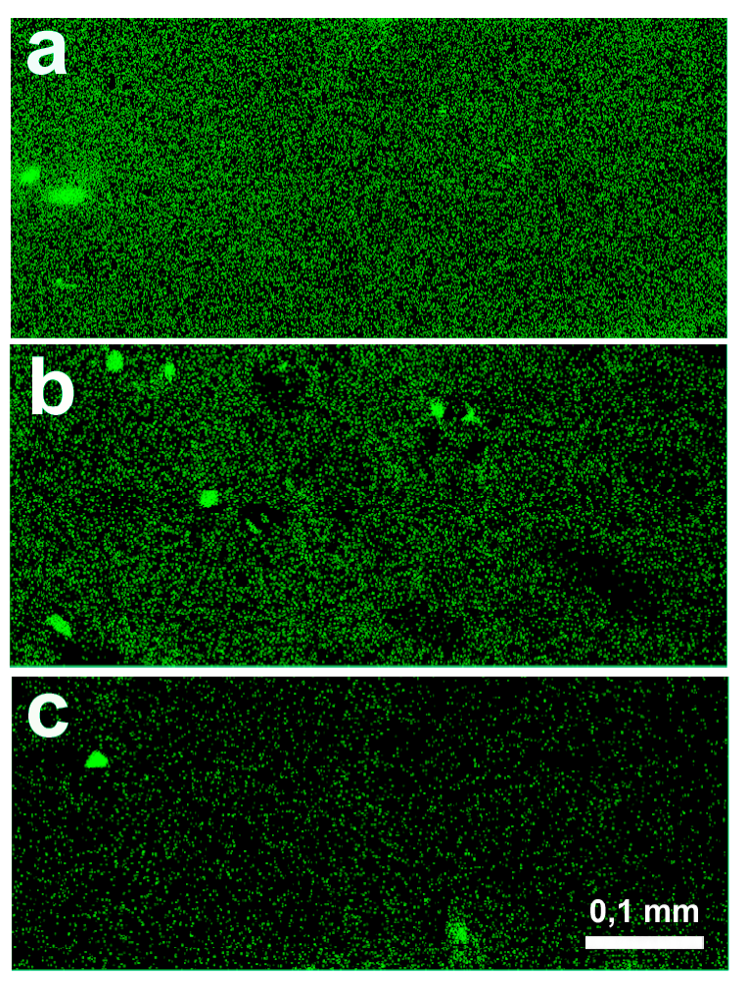
Detection of anti-glycosaminoglycan (GAG) antibody binding to the extracellular matrix of hyaline cartilage. Microphotograph **(a) **shows the binding of rheumatoid arthritis (RA) serum-derived antibodies to human cartilage. The reactivity could be inhibited by pre-incubation of the serum sample with chondroitin sulphate (CS) C. **(b) **is using 2 mg/ml CSC; **(c) **is using 4 mg/ml CSC as an inhibitor). Magnification: 100×

Finally, we tested the ability of anti-GAG antibodies to bind to the extracellular matrix of hyaline cartilage. The relatively strong binding of serum antibodies to the extracellular matrix of hyaline cartilage could be increasingly inhibited by pre-incubation of the serum with increasing amounts of CSC (Figure 4).

## Discussion

Several lines of evidence support the role of B-cells in the pathology of RA. The RA synovium is populated by cells that produce RFs, anti-type II collagen and anti-CCP antibodies. Furthermore, B-cell depletion by the anti-CD20 monoclonal antibody rituximab decreases disease activity and provides prolonged remission [23], further demonstrating that B-cell activation is important in the pathogenesis of RA.

The existence of carbohydrate-specific antibodies has long been known, with examples including the blood group-specific antibodies described by Karl Landsteiner [24]. Some of the carbohydrate-specific antibodies have also been implicated in the pathomechanism of autoimmune diseases, e.g. in rheumatic carditis [25]. Surprisingly, carbohydrate-specific antibodies have not yet been systematically investigated in RA, in spite of the fact that hyaline cartilage and synovial fluid contain large amounts of GAGs including HA, CS and KS. These carbohydrates are degraded and released from the tissue during the course of joint inflammation [26,27].

In this study we found that while GAG-specific antibodies were absent in neonates, they were present in high amounts in sera of adults. The repetitive nature of GAGs and the absence of anti-GAG antibodies in neonates thus, places GAGs in the category of TI2 antigens [28-31]. Hence, B1 B cells, known to be reactive to TI2 antigens, are the most probable sources of anti-GAG nAbs.

As GAG-specific antibody binding was inhibited by an anionic exchanger resin, we hypothesise that weak ionic interactions might play a pivotal role in the recognition of polyanionic GAGs by these immunoglobulins. In line with the polyreactive nature of nAbs, we found a strong statistical correlation among IgM-type GAG-specific antibodies. Also, in inhibition studies we have shown broad cross-reactivity among different types of GAGs. This provides further support for the NAb nature of the detected anti-GAG antibodies. There was much less correlation among IgG-type GAG-specific antibodies, raising the possibility that these IgG antibodies might represent a distinct population that is produced after class switch recombination, that could also possibly require T-cell help. Recently, a specific set of T-cells have been reported to recognise GAGs both in a murine model of arthritis and in patients with RA [32]. Thus, the fully T-independent nature of anti-GAG antibodies remains to be elucidated.

In the present work we have shown that all IgM anti-GAG antibodies and certain IgG-type anti-GAG antibodies were significantly elevated in sera of patients with RA compared with adult controls. These anti-GAG antibodies of RA patients were not only present in the systemic circulation and in the synovial fluid, but they were also fully capable of binding to the extracellular matrix of hyaline cartilage. Synovial fluid/serum anti-GAG antibody ratios were consistently low for IgM antibodies and were relatively high in the case of IgGs. In contrast to IgM, IgG gets into the inflamed synovial fluid readily, and thus, our data argue against local production of IgM type anti-GAG antibodies.

It may be hypothesised that the anti-GAG antibody production is up-regulated in RA possibly because of the extensive release of cartilage molecules. Autoantigens are continuously present in large amounts in the body. They do not delete B1 Bcells and, n the presence of alarm signals, autoantigens may even efficiently stimulate them. In contrast to the B1a B-cell population (that shows a constitutive low activity), CD5 (-) B1b B-cells (implicated in the synthesis of TI2 antigen-specific antibodies) were shown to be capable of significant increases in their activity [33,34].

The next important step was to test if the levels of anti-GAG antibodies showed any correlation with the disease activity in RA. Intriguingly, using a multistep approach, our work has demonstrated that CSC-specific IgM antibody levels show a clear inverse correlation with the activity of RA. Thus, we suggest that CSC-specific IgM is a disease-state biomarker in RA. We found a similar relation when we analysed the connection between disease activity, CRP and anti-CSC IgM concentrations. We hypothesise that during the active phase of the disease, the levels of anti-GAG antibodies are reduced because of binding of these antibodies to GAGs released from the degrading cartilage. This could also explain the significantly reduced level of synovial fluid anti-GAG antibodies as compared with the one measured in the serum of patients with RA. The absence of GAG-specific antibodies in neonates and the abundance of them in adults, as well as their highly cross-reactive nature, raise the possibility that anti-GAG antibodies are produced on exposure to environmental microbial carbohydrates or glycosylated antigens. Thus, we next assessed if GAG-specific antibodies could cross-react with microbial antigens. We found that circulating anti-GAG antibodies were capable of binding to bacterial peptidoglycans and the fungal polysaccharide, Zymosan. Such cross-reactions may not only explain the absence of anti-GAG antibodies in neonates, but may also link infectious agents and RA.

To further address whether microbial infections could contribute to carbohydrate-specific immune responses, we investigated reactivity of serum antibodies to glycosidase-digested aggrecan, a major macromolecule of hyaline cartilage that carries over 100 GAG side chains. We found a significantly increased antibody recognition of aggrecan modified in its GAG chains by either β-galactosidase or hyaluronidase digestion. Hyaluronidase is a virulence factor of *S. aureus*, *Streptococcus pyogenes *or *Clostridium perfringens *[35-37], while b-galactosidase is expressed by *E. coli *[38]. Therefore it is very likely that carbohydrate neoepitopes are generated *in vivo *locally by the activity of these glycosidases during infections. An increasing body of evidence suggests that cell surface carbohydrate pattern is important in immune recognition and self/non-self discrimination [39]. As glycosidases modify GAG side chains of proteoglycans, they can also alter the glycosylation pattern in the tissues. Using a carbohydrate array, we attempted to characterise the carbohydrate-specific serum and synovial fluid IgG reactivity patterns of patients with RA and controls. However, by using a currently available carbohydrate array, we could not identify a disease-specific carbohydrate recognition pattern. Arrays with more complex display of carbohydrate structures would be necessary to gain an insight into the exact epitope specificity of the circulating carbohydratespecific antibodies.

While anti-GAG antibody levels are increased in RA, probably because of the increased efflux of GAGs from the inflamed joint, higher anti-GAG levels are associated with less severe disease according to our present data. The mechanism of the "protective" role of anti-GAG antibodies is not known. Both peptidoglycans and HA and HS fragments have been shown to bind to certain toll-like receptors (TLRs) [39]. Our hypothesis is that one of the roles of the natural anti-GAG antibodies is to bind to the degrading matrix molecules and thus, prevent the excessive ligation of danger receptors like TLRs. Hence, the NAbs may reduce the chance of priming of naive T-cells to self molecules. Also, the degrading matrix releases not only pure GAG fragments but also GAGs coupled to peptides of proteoglycans undergoing proteolysis. While carbohydrates by themselves may not elicit a strong immune response, glycopeptides are often strongly immunogenic (as clearly exemplified by conjugate vaccines [40]). Therefore, by masking GAG epitopes and facilitating the elimination of such glycopeptides (possibly in the form of immune complexes), anti-GAG antibodies may have an important function in preventing or decelerating the autoimmune processes.

## Conclusion

It has been proposed that changes in the global NAb network (referred to as "immunculus distortions") may serve as predictors or early disease markers [41]. Inthe current work we found that anti-GAG antibody levels sharply discriminate between adult controls and RA patients with low disease activity (DAS 28 ≤ 3.2), and thus, anti-GAG antibodies may be the key to an inexpensive early disease activity biomarker. Thus, they might have the justification to be incorporated into autoantigen microarrays.

## Abbreviations

ACR: American Colleage Rheumatology; ACPA: anti-citrullinated protein antibody; BSA: bovine serum albumin; CCP: cyclic citrullinated peptide; CRP: C-reactive protein; CS: chondroitin sulphate; DAS: disease activity score; GAG: glycosaminoglycan; HA: hyaluronic acid; HS: heparan sulphate; KS: keratan sulphate; Nab: natural autoantibody PBS: Phosphate buffered saline; PGIA: proteoglycan-induced arthritis, aggrecan induced arthritis RA: rheumatoid arthritis; RF: rheumatoid factor.

## Competing interests

The authors declare that they have no competing interests.

## Authors' contributions

BG, LT, GN, MP, AF and EIB participated in the design of the study. Experiments were performed by BG, ZL, PP, PM and AK. Statistical analysis was carried out by TL. GN, AP, BR, IU, GP and PG contributed by providing human samples. Analysis of data was carried out by BG, TL, GN, MP, AF and EIB. Intellectual contributions to the manuscript were provided by BG, LT, GN, MP, WZ, AF and EIB. All authors read and approved the final manuscript.
